# An interpretable machine-learning model for predicting in-hospital mortality in patients with sepsis-associated acute kidney injury

**DOI:** 10.3389/fmed.2026.1756831

**Published:** 2026-02-13

**Authors:** Jian-Zhong Wang, Nan Zhang, Rong-Rong Ma, Mei Yang, Yao-Geng Chen, Wen-Jie Zhou

**Affiliations:** 1 School of Clinical Medicine, Ningxia Medical University, Yinchuan, China; 2 General Hospital of Ningxia Medical University, Yinchuan, China; 3 Ningxia Medical University, Yinchuan, China

**Keywords:** acute kidney injury, critical illness, machine learning, MIMIC-IV database, sepsis

## Abstract

**Introduction:**

Sepsis-associated acute kidney injury (S-AKI) is a severe complication in critically ill patients, linked to increased short-term mortality and chronic kidney disease. Existing prognostic tools like SOFA and SAPS II lack full representation of intricate clinical variable interactions. Machine learning (ML) models have potential in intensive care, but few validated and interpretable models focus on the in-hospital mortality rate of patients with S-AKI. This study aims to create and validate ML models for forecasting in-hospital mortality in S-AKI patients, identifying the most effective predictive model.

**Methods:**

We conducted a retrospective analysis of data from the MIMIC-IV 3.0 database to identify adult ICU patients who met theSepsis-3.0(Sepsis-3 was defined as suspected infection with an acute increase in SOFA score ≥2) and KDIGO criteria for S-AKI. Additionally, a prospective cohort study from the General Hospital of Ningxia Medical University spanning 2023 to 2025 was included. Predictors recorded within 24 h of ICU admission included demographic information, comorbidities, vital signs, laboratory results, treatments, and severity scores. Variables with more than 20% missing data were excluded, and the remaining data were processed using interpolation. Feature selection was performed using the Boruta algorithm, and five machine learning models were trained (XGBoost, Random Forest, LightGBM, Decision tree, logistic regression). Model performance evaluation was based on metrics such as AUC, accuracy, sensitivity, specificity, F1 score, and clinical efficacy assessed through decision curve analysis. To enhance model interpretability, the SHapley Additive exPlanations (SHAP) method was employed.

**Results:**

Among 16,800 patients with S-AKI, non-survivors(in-hospital mortality) exhibited older age, higher disease severity scores, more pronounced fluid overload, poorer renal function, metabolic acidosis, coagulation disorders, and heightened inflammatory responses. The XGBoost model demonstrated superior discriminative power (AUC 0.8799 置信区间) in internal validation, surpassing other ML models, with exceptional sensitivity, accuracy, and F1 score. Decision curve analysis revealed that LightGBM offered the most significant net clinical benefit across various threshold probabilities. SHAP analysis consistently identified SAPS II score, AKI stage, oxygenation index, and key biochemical markers (e.g., serum sodium and blood urea nitrogen) as primary contributors to mortality risk, while the added value of basic demographic variables was limited. External validation confirmed that the XGBoost model has potential discrimination and robustness, highlighting the robustness and wide applicability of the machine learning-based prognostic framework.

**Conclusion:**

This study established an externally validated and interpretable ML model for riskstratification in S-AKI, enabling early identification of high-risk patients, personalized management strategies, and enhanced clinical outcomes in sepsis care.

## Background

Sepsis (1), a life-threatening condition characterized by host response dysregulation due to infection, is a major cause of mortality in global intensive care units (ICUs). Approximately 48.9 million sepsis cases occur annually worldwide, with a mortality rate nearing 20% (2). Among the various complications of sepsis, sepsis-associated acute kidney injury (S-AKI) stands out, affecting up to 50% of ICU sepsis patients (3). S-AKI significantly raises in-hospital mortality by 6 to 8 times and triples the likelihood of developing chronic kidney disease (CKD) (2). The pathophysiology of S-AKI involves a complex interplay of hemodynamic instability, inflammation, and microvascular dysfunction, often leading to rapid deterioration and posing challenges for timely intervention (4). Hence, early prognosis prediction for S-AKI is vital for risk assessment, personalized treatment approaches—such as optimizing fluid administration, employing vasopressors, or initiating renal replacement therapy—and potentially mitigating adverse outcomes. This underscores the critical need to enhance prognostic tools in this domain to address the clinical urgency associated with S-AKI management (5).

Despite advancements in the understanding of S-AKI, traditional risk assessment methods, including scoring systems like the Sequential Organ Failure Assessment (SOFA) and the Simplified Acute Physiology Score II (SAPS II), frequently fail to account for the multifaceted and nonlinear interactions among clinical variables, leading to inadequate prediction accuracy (6). Machine learning (ML) algorithms have emerged as a transformative approach in critical care medicine, utilizing large datasets to discern subtle patterns and enhance prognosis modeling (7). Research indicates that ML models, such as XGBoost and Random Forest, surpass traditional methods in predicting AKI events and mortality in sepsis cohort studies. By incorporating electronic health record data, these models achieve an area under the receiver operating characteristic curve (AUC) value exceeding 0.85. These developments underscore the potential of ML, particularly in elucidating feature contributions and improving clinical applicability through interpretable technologies like SHapley Additive exPlanations (SHAP) (8). Nonetheless, gaps remain in the external validation of models for S-AKI prognosis, particularly those that incorporate real-world heterogeneous data from diverse populations to ensure generalizability.

The advancement of big data and artificial intelligence technologies has enabled machine learning (ML) to excel in developing critical illness prognosis models by effectively managing intricate nonlinear and high-dimensional features (9). A predictive model was developed to evaluate the risk of acute kidney injury (AKI) in sepsis patients by leveraging the MIMIC-III database. The model incorporated Boruta feature selection and various machine learning (ML) algorithms. The XGBoost model achieved an AUC of 0.821, surpassing the performance of both SOFA and SAPS II scores, highlighting the potential of ML in early detection of sepsis-induced renal impairment. In a study focusing on elderly AKI patients, LightGBM models were constructed to forecast the onset of AKI and in-hospital mortality using data from MIMIC-IV and an external dataset. These models exhibited robust discrimination and calibration across training and validation datasets (10). Additionally, they visually illustrated the significant contribution of key features (e.g., 3-day creatinine, Sepsis, BUN changes, and blood pressure) to the model output through the SHAP method. Moreover, they developed an online accessible network tool based on the model, offering a novel approach for clinical intelligent decision-making.

In this study, the latest version of the mimic-IV 3.0 database was used to include patients with Sepsis-3 and KDIGO (11) criteria for sepsis-related acute kidney injury (S-AKI). Aiming to develop and validate multiple machine learning models to predict the mortality rate of acute kidney injury in patients with sepsis, we acknowledged potential machine learning-related biases (such as selection bias, leakage, and overfitting). Therefore, we also included an external validation cohort prospectively collected by the Department of Critical Care Medicine of General Hospital of Ningxia Medical University from 2023 to 2025. Combining external validation and pre-defined sensitivity analysis, the model with the optimal predictive performance was determined.

## Methods

### Training cohort

The MIMIC-IV Database 3.0 (12–14), an open and free intensive care database, represents the latest iteration and encompasses comprehensive clinical data for inpatients at Beth Israel Deaconess Medical Center from 2008 to 2019. MIMIC-IV serves as an updated version of MIMIC-III, incorporating contemporary data and enhancing several aspects of its predecessor. It includes records for 200,000 emergency patients and 70,000 ICU patients. The clinical data within the database comprises demographic characteristics, vital signs, imaging examinations, laboratory test results, data dictionaries, and files containing codes from the ninth and tenth editions of the International Classification of Diseases (ICD-9 and ICD-10). The hourly physiological data are recorded from adjacent monitors and verified by ICU nurses. Since the health information derived from the MIMIC-IV database is de-identified, informed consent from patients is not required (10, 15).

The inclusion criteria comprised patients admitted to the ICU for the first time and diagnosed with sepsis according to the 9th Edition of the International Classification of Diseases (ICD-9) codes (99,591, 99,592, 78,552). Acute kidney injury (AKI) was assessed and classified based on the highest serum creatinine (SCr) level and urine output as defined by the Global Nephropathy Improvement Prognosis (KDIGO). The criteria for AKI are as follows: an increase in SCr to ≥1.5 times the baseline must occur within the first 7 days; an increase in SCr of ≥0.3 mg/dL within 48 h; or urine output of less than 0.5 mL/kg/h for 6 h or longer. If the SCr prior to admission is not documented, the initial SCr value at admission is considered the baseline SCr. In this study, AKI was evaluated based on the worst serum creatinine and urine output recorded within 72 h following the diagnosis of suspected sepsis.

Exclusion criteria are as follows: (1) Length of stay in the ICU is less than 48 h; (2) Age is less than 18 years or greater than 89 years; (3) Presence of pre-existing acute kidney injury (AKI) or chronic renal failure; (4) Missing data exceeds 20%; (5) The patient has received renal replacement therapy (RRT) or continuous renal replacement therapy (CRRT) upon admission.

### External validation cohort

Patients were enrolled in the Intensive Care Unit of the General Hospital of Ningxia Medical University from 2023 to 2025. The study included adult patients diagnosed with S-AKI. The exclusion criteria were identical to those applied in the training cohort. This study received approval from the Ethics Committee of the General Hospital of Ningxia Medical University.

### Data extraction

Patient data within the first 24 h post-admission were extracted from the MIMIC-IV repository. The study incorporated the following data categories: (1) Demographic attributes encompassing gender, age, and race; (2) Comorbidities such as congestive heart failure, hypertension, chronic lung disease, diabetes, and liver disease; (3) Physiological parameters comprising heart rate, body temperature, blood oxygen saturation (PO2), systolic blood pressure (SysBP), and diastolic blood pressure (DiasBP); (4) Laboratory metrics encompassing total bilirubin, anion gap, albumin, chloride, potassium, sodium, lactic acid, partial thromboplastin time (PTT), prothrombin time (PT), international normalized ratio (INR), creatinine, blood urea nitrogen (BUN), and blood glucose; (5) Therapeutic interventions and clinical strategies including mechanical ventilation and vasopressor administration. In cases where variables had multiple measurements, both the maximum and minimum values were included for analysis. Initial test values were exclusively considered for the analysis of SOFA and SAPS-II scores. As this study is hypothesis-driven, no efforts were made to determine the sample size; instead, all eligible patients in the MIMIC-IVI database were encompassed to optimize statistical power.

To minimize bias resulting from missing data, variables with missing values exceeding 20% are excluded from the final analysis, while the remaining variables undergo multiple imputation (MI). MI is a robust and widely employed technique for addressing missing values (16). This method assigns multiple plausible values to each missing entry, accounting for the uncertainty associated with these gaps. Consequently, several datasets can be generated, allowing for the estimation of parameters of interest (17). For example, if the focus is on the coefficients of a specific covariate within a multivariable model, the coefficients can be estimated from each dataset, resulting in multiple coefficient values. Hyperparameters are searched and determined within the training set through K-fold cross-validation. Category imbalance is handled through class_weight; The final list and quantity of Boruta features can be found in the Supplementary materials. By considering the uncertainty inherent in missing value estimation, these coefficients are then combined to yield a more accurate overall estimate. The variance of coefficients estimated through MI is less likely to be underestimated compared to that derived from a single imputation approach (18).

### Statistical analysis

Statistical analysis, modeling, and verification were conducted using Python 3.8 software and associated module packages (19). The training/validation division adopted stratified sampling based on in-hospital mortality outcomes to maintain consistent category ratios. Hyperparameters are completed within the training set through cross-validation, and the validation set is only used for the final evaluation. Normally distributed continuous variables are presented as mean ± standard deviation, while non-normally distributed variables are expressed as median (quartile range). Categorical variables are represented as percentages. In univariate analysis, categorical variables are compared using the Pearson chi-square test or Fisher’s exact test, whereas continuous variables are compared using the Student’s *t*-test or the Kruskal–Wallis test. A *p* value of less than 0.05 is deemed statistically significant. Internal validation employed the area under the ROC curve (AUC), accuracy, precision, and F1 score to evaluate the performance of models developed by five machine learning algorithms, with the model demonstrating the best performance selected as the final prediction model. The results of the optimal prediction model are explained using Shapley additive explanation (Shap) (20).

When the Shap value of a variable in the sample exceeds 0, it indicates that the variable positively influences the prediction of the outcome at that moment. To assess how each variable impacts the prognosis of SA-AKI patients during hospitalization, we generated the Shap summary plot and Shap dependency plot for the final prediction model. These visualizations reveal how the positive and negative effects of each variable on outcome prediction fluctuate with its value. Additionally, the Shap maps for the patients were created to illustrate how the model personalizes predictions for each patient’s condition and informs clinical decision-making.

## Results

### Baseline characteristics

A total of 16,800 patients participated in this study, with 13,455 in the survival group and 3,345 in the non-survival (in-hospital mortality) group. First of all, we state: Baseline differences mainly reflect severity associated with mortality; our model is intended for prognostic risk stratification rather than causal inference. All predictor variables were limited to being obtained within the first 24 h (or the preset window) after ICU admission. We assessed calibration using reliability curves and Brier scores in both internal and external validations. Compared to the survival group, patients in the non-survival group were significantly older and had a higher proportion of females. The SAPS II and SOFA scores were markedly elevated in the non-survival group. During hospitalization, a greater proportion of patients in the non-survival group received continuous renal replacement therapy (CRRT), and the use of norepinephrine also increased significantly. Although fluid intake was slightly lower in the non-survival group, urine output was significantly reduced. Nonetheless, the overall fluid balance was higher in the non-survival group, indicating more pronounced fluid retention. Concurrently, serum creatinine and blood urea nitrogen levels were significantly elevated, while total carbon dioxide and pH values were lower. Potassium ion concentration was higher, and chloride ion levels were slightly lower than those in the survival group, with no significant difference in sodium ion levels. These findings reflect the worsening of renal function and a trend toward metabolic acidosis.

Coagulation indicators, including PT, PTT, and INR, were significantly elevated in the non-survival group (all *p* < 0.001), indicating a more severe coagulation disorder. Additionally, white blood cell count, lactic acid levels, red blood cell distribution width, and blood glucose concentrations were all significantly increased, reflecting poor tissue perfusion and an intensified inflammatory response. In contrast, the observed low hemoglobin and red blood cell counts suggest a deterioration in the patient’s inflammatory response and coagulation disorders.

The non-survival group exhibited a higher heart rate, slightly lower body temperature, a mild decrease in systolic blood pressure, and a significant reduction in blood oxygen saturation, indicating compromised circulatory and oxygenation functions. Diabetes and hypertension were slightly more prevalent in the survival group, whereas myocardial infarction was more common in the non-survival group. Overall, patients in the non-survival group demonstrated elevated disease severity scores, impaired renal function, significant metabolic acidosis, inflammatory responses, and coagulation disorders, coupled with hypotension, hypothermia, and hypoxemia. These findings collectively suggest that multiple organ dysfunction and circulatory instability are the primary contributors to mortality.

### Function selection

**Figure 1:**
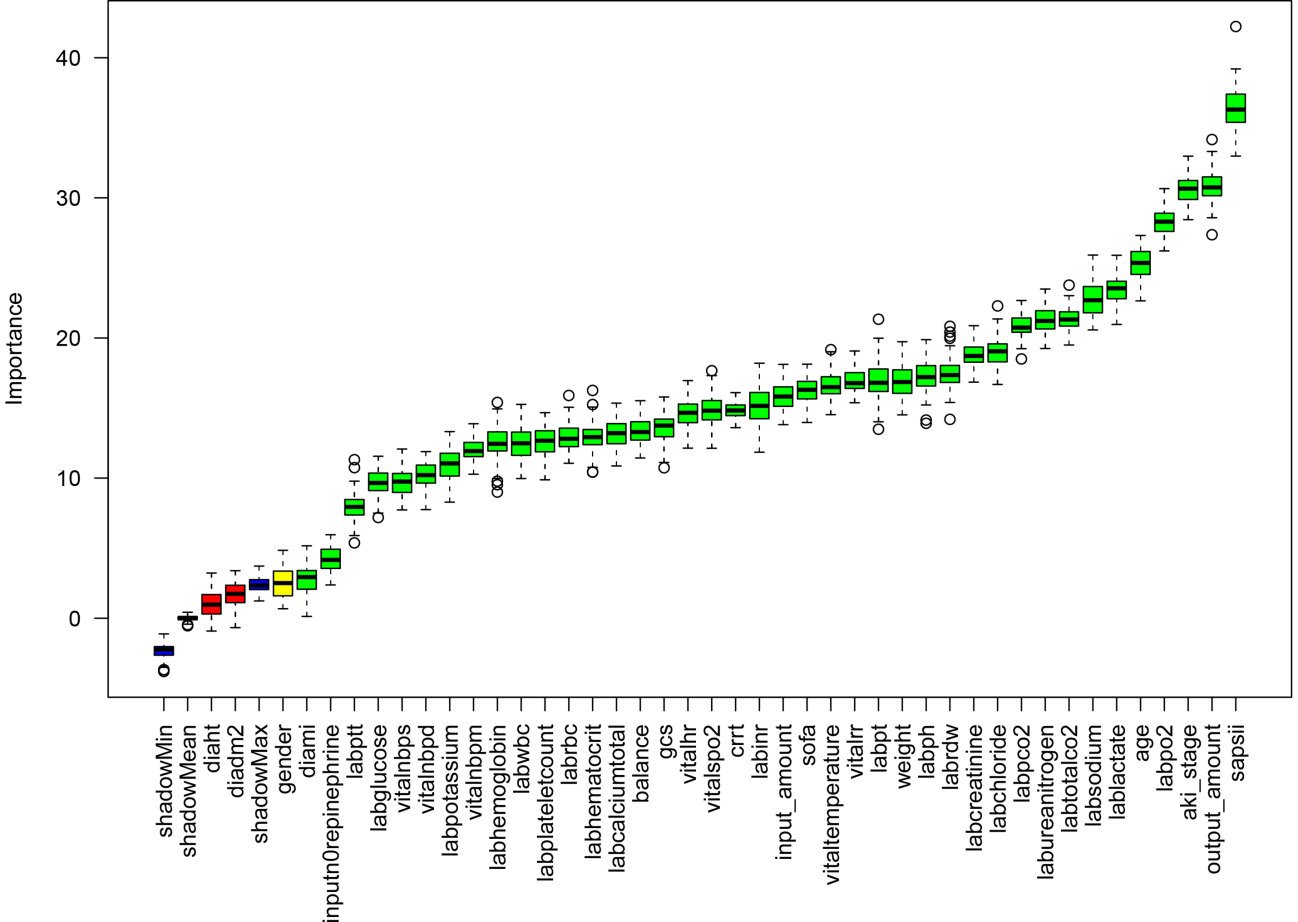
Feature selection based on the Boruta algorithm. The horizontal axis is the name of each variable, and the vertical axis is the *Z*-value of each variable. The box plot shows the *Z*-value of each variable during model calculation. The green boxes represent the first 41 important variables, the yellow represents tentative attributes, and the red represents unimportant variables.

The Boruta algorithm, an effective tool for feature selection and importance ranking, is employed in this study to evaluate candidate variables within the context of a random forest model. The results of this analysis are visually depicted in Figure 1. These findings illustrate a distinct hierarchical distribution of variable importance within the model.

The primary important variables consist of the SAPS II score, AKI stage, age, serum sodium (labsodium), blood urea nitrogen (labureanitrogen), and serum chloride (labchloride). Secondary variables comprise body weight, blood gas pH, coagulation-related indicators (PT, INR), SOFA score, heart rate, and GCS score. Tertiary variables include blood pressure parameters, hemoglobin, red blood cells, total calcium, gender, height, history of hypertension, and imaging findings. Certain variables exhibit a negative distribution. In summary, the model outcomes suggest that indicators reflecting disease severity, physiological burden, and renal function status (e.g., SAPS II, AKI stage, serum sodium, blood urea nitrogen) are the primary determinants, whereas the predictive utility of basic demographic characteristics (gender, height) and some routine laboratory indicators is constrained (see Table 1).

**Table 1:** Baseline characteristics of the patients with sepsis.

Variables	No (*N* = 13,455)	Yes (*N* = 3,345)	*p*
sapsii	39.1 ± 12.9	49.2 ± 14.7	<0.001
aki_stage			<0.001
1	3,505 (26%)	354 (10.6%)	
2	6,954 (51.7%)	1,279 (38.2%)	
3	2,996 (22.3%)	1712 (51.2%)	<0.001
output_amount	2640.5 ± 3445.7	1894.5 ± 2789.3	<0.001
labpo2	150.2 ± 76.8	111.5 ± 59.4	<0.001
age	66.2 ± 15.8	71.7 ± 15.0	<0.001
lablactate	2.1 ± 1.2	2.8 ± 2.3	<0.001
labsodium	138.5 ± 4.6	138.5 ± 6.2	0.369
labrdw	15.0 ± 2.1	16.2 ± 2.7	<0.001
labureanitrogen	26.3 ± 20.3	37.9 ± 26.8	<0.001
weight	86.6 ± 25.6	79.6 ± 23.3	<0.001
vitaltemperature	36.8 ± 1.7	36.7 ± 2.7	0.001
labtotalco2	25.0 ± 4.3	23.8 ± 5.6	<0.001
labph	7.4 ± 0.1	7.4 ± 0.1	<0.001
sofa	5.7 ± 3.3	7.6 ± 4.1	<0.001
labpt	15.9 ± 6.6	18.9 ± 9.9	<0.001
labchloride	104.9 ± 5.9	103.7 ± 7.5	<0.001
vitalrr	26.1 ± 781.1	20.9 ± 4.8	0.728
labinr	1.5 ± 0.7	1.7 ± 1.0	<0.001
labpco2	41.4 ± 7.8	40.6 ± 9.7	<0.001
vitalhr	86.0 ± 15.7	89.8 ± 17.7	<0.001
crrt			<0.001
No	12,810 (95.2%)	2,846 (85.1%)	
Yes	645 (4.8%)	499 (14.9%)	
inputn0repinephrine			<0.001
No	10,468 (77.8%)	2062 (61.6%)	
Yes	2,987 (22.2%)	1,283 (38.4%)	
labptt	37.5 ± 16.7	43.0 ± 21.4	<0.001
labwbc	12.9 ± 8.4	14.7 ± 12.3	<0.001
labglucose	141.5 ± 52.9	151.4 ± 63.1	<0.001
input_amount	5223.2 ± 3647.4	4812.9 ± 3857.4	<0.001
diadm2			0.049
No	9,420 (70%)	2,400 (71.7%)	
Yes	4,035 (30%)	945 (28.3%)	
labcreatinine	1.5 ± 1.5	1.8 ± 1.5	<0.001
Gender			<0.001
Female	5,452 (40.5%)	1,499 (44.8%)	
male	8,003 (59.5%)	1846 (55.2%)	
diaht			<0.001
No	7,376 (54.8%)	2056 (61.5%)	
Yes	6,079 (45.2%)	1,289 (38.5%)	
diami			<0.001
No	12,566 (93.4%)	2,987 (89.3%)	
Yes	889 (6.6%)	358 (10.7%)	
labrbc	3.5 ± 0.7	3.4 ± 0.7	<0.001
labplateletcount	194.4 ± 101.9	195.7 ± 119.0	0.515
labhemoglobin	10.6 ± 1.9	10.3 ± 2.1	<0.001
labhematocrit	32.0 ± 5.6	31.6 ± 6.2	<0.001
labpotassium	4.2 ± 0.6	4.3 ± 0.7	<0.001
labcalciumtotal	8.2 ± 0.7	8.2 ± 0.8	0.376
Balance	2586.2 ± 4569.9	2921.3 ± 4535.9	<0.001
vitalnbps	115.8 ± 16.6	114.5 ± 72.1	0.002
vitalnbpd	63.9 ± 28.5	64.3 ± 68.1	0.632
vitalnbpm	82.8 ± 694.2	78.2 ± 147.5	0.723
vitalspo2	102.5 ± 438.9	96.7 ± 7.6	<0.001
gcs	13.3 ± 3.1	12.8 ± 3.4	<0.001

### Model performance comparison

**Figure 2:**
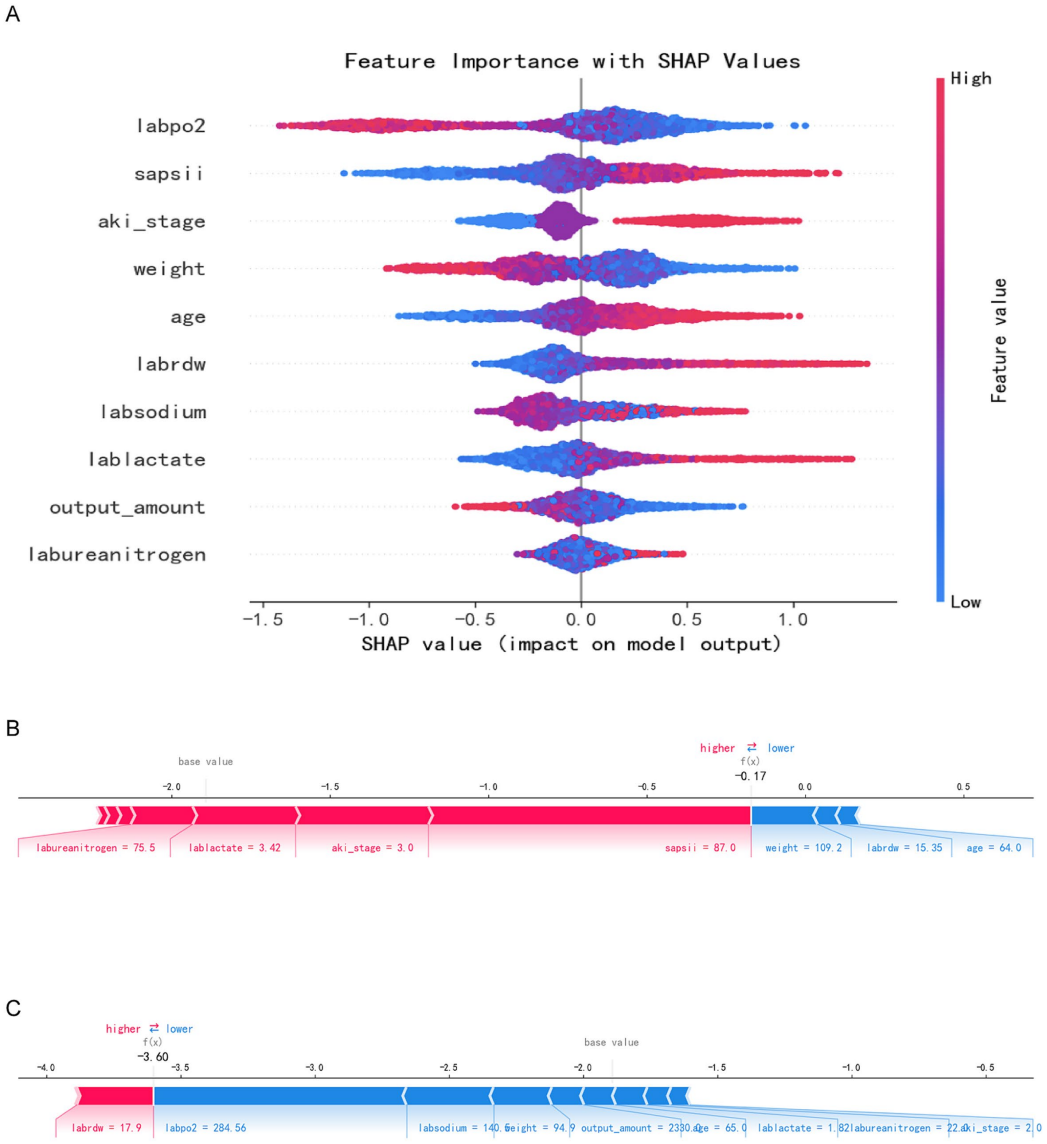
SHAP analysis results. **Panel A:** SHAP summary plot with feature importance values (e.g., labpo2, sapsii, aki_stage). **Panels B and C:** Individual SHAP value plots for two specific predictions.

**Figure 3:**
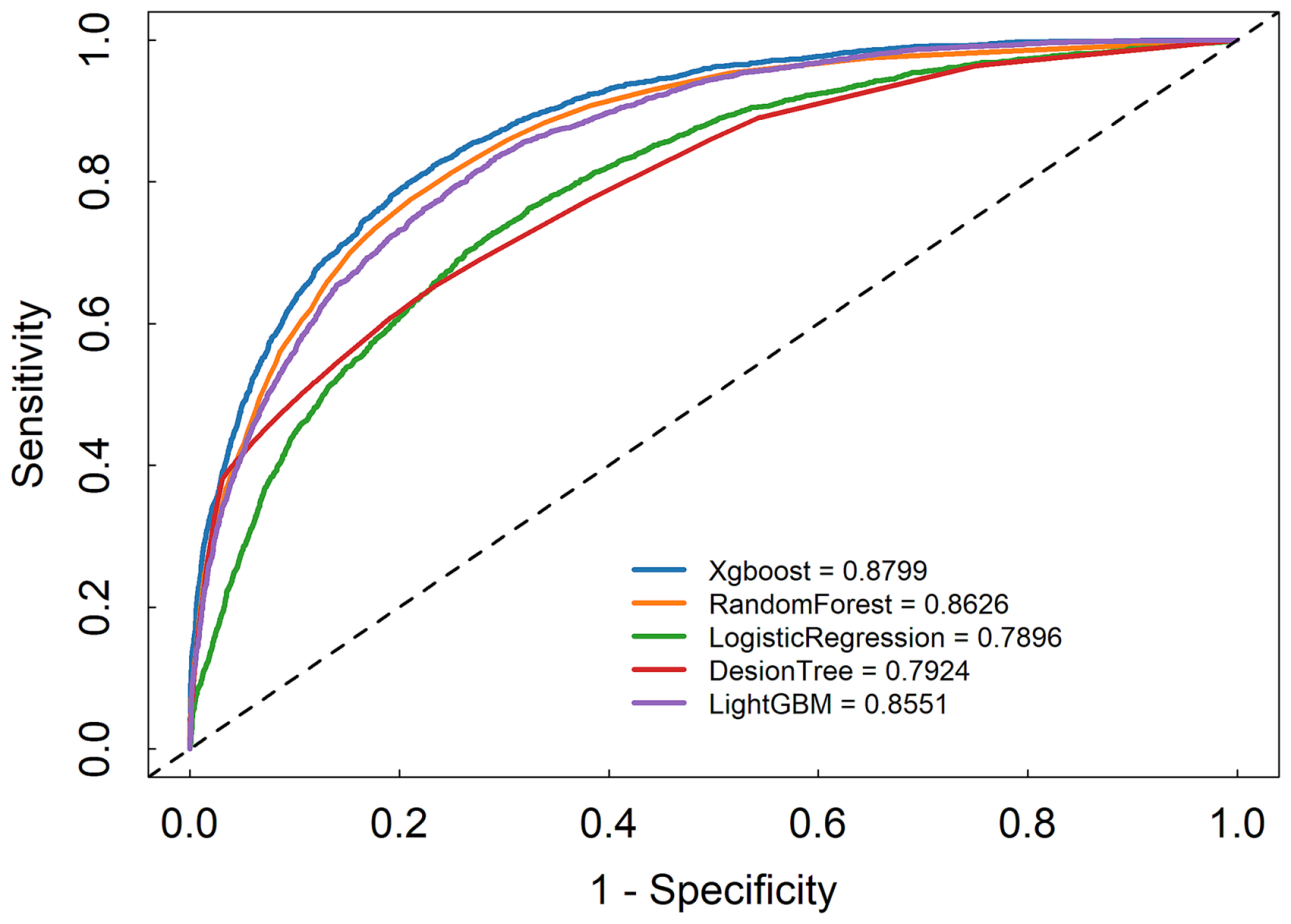
Receiver operating characteristic curve of the seven models.

**Figure 4:**
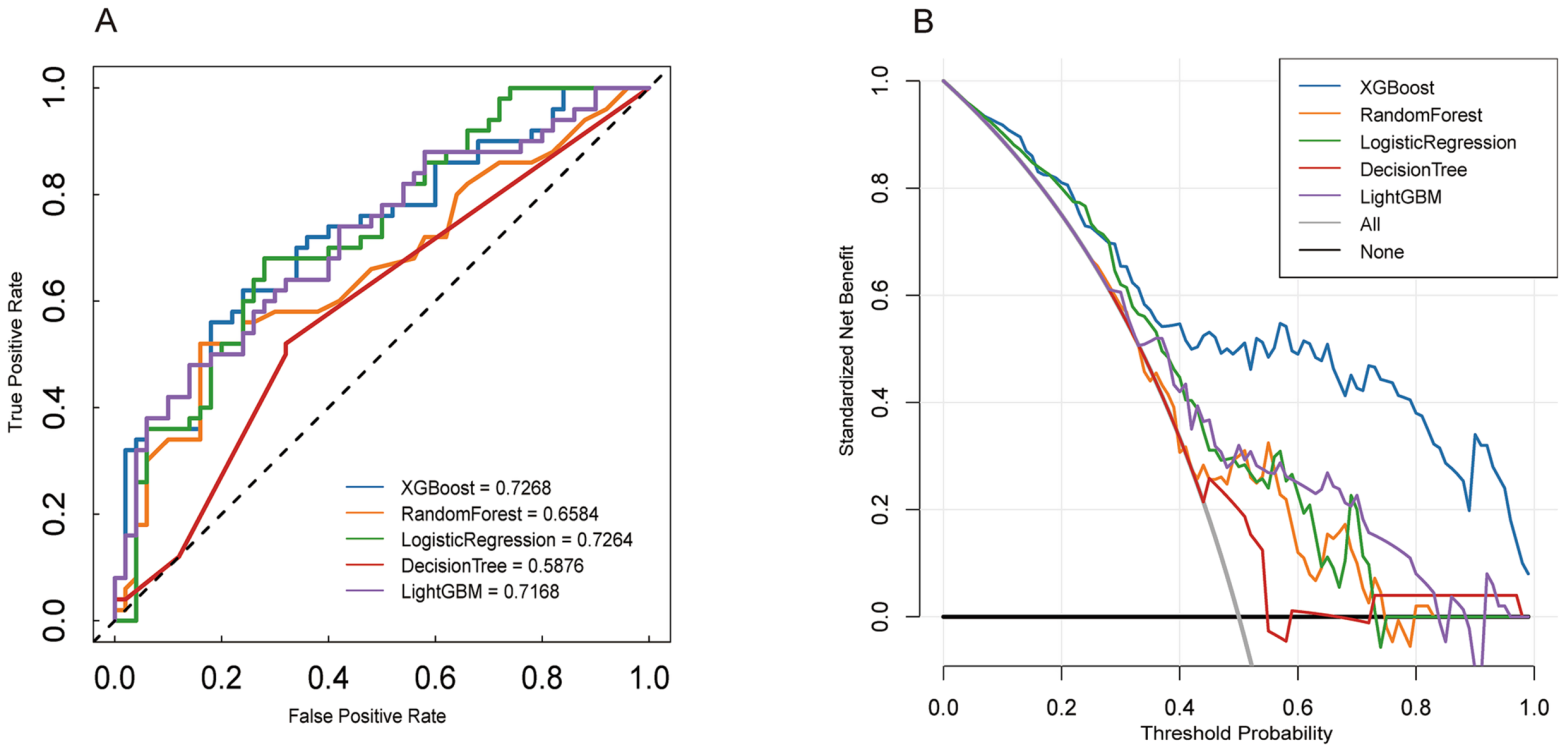
External verification Model performance evaluation. **Panel A:** Receiver Operating Characteristic (ROC) curves comparing the performance of XGBoost, Random Forest, Logistic Regression, Decision Tree, and LightGBM models. **Panel B:** Decision curve analysis for the same models.

We developed five machine learning (ML) models to predict the onset of acute kidney injury (AKI) in patients with sepsis following their admission to the intensive care unit (ICU). Figure 2 illustrates the discriminatory performance of these models, as represented by the receiver operating characteristic (ROC) curve. Among the five models, the XGBoost model exhibited the highest predictive accuracy for patients in the sepsis survival group, with an area under the curve (AUC) of 0.8799. This was followed by the random forest model (AUC = 0.8626), LightGBM (AUC = 0.8551), decision tree (AUC = 0.7924), and logistic regression (AUC = 0.7896) (Figure 3). Table 2 presents a comprehensive set of performance indicators for the five models. The XGBoost model demonstrates superior discriminative power, achieving the highest sensitivity (0.822), accuracy (0.787), and F1 score (0.606), along with the third-highest specificity (0.778). According to the decision curve analysis (DCA) curve shown in Figure 4, the LightGBM model offers a greater net benefit and threshold probability compared to the other models, suggesting that it is the optimal choice with significant clinical applicability. We report the calibration curve with the Brier score to evaluate the reliability of the predicted probability. Meanwhile, when the predicted risk is greater than or equal to the preset threshold, enhanced monitoring and early renal support assessment can be considered.

**Table 2:** Model performance metrics.

Model	AUC	Accuracy	F1	Sensitivity	Specificity
XGBoost	0.8799	0.787	0.606	0.822	0.778
RandomFor	0.8626	0.77	0.584	0.811	0.76
LogistickR	0.7896	0.692	0.5	0.772	0.672
DecisionT	0.7924	0.757	0.508	0.629	0.789
LightGBM	0.8551	0.765	0.577	0.804	0.755

### Explanation of risk factors

The Shap summary chart illustrates the impact of each baseline variable on the prognosis of SA-AKI. Each patient’s characteristics are depicted as individual points, colored according to their attribute values, with red indicating higher values and blue representing lower values, as demonstrated in the Shap summary graph (Figure 2A). A higher Shap value for a baseline variable correlates with an increased probability of death during hospitalization. Each point on the Shap dependency graph signifies a patient, revealing how the importance of the baseline variable fluctuates with changes in its value. The risk of death during hospitalization is elevated when the Shap value exceeds zero. Figures 2B,C each illustrate distinct sample sets showcasing risk and protective factors denoted by red and blue attributes. In Figure 2B, a high-risk scenario is evident where the presence of red characteristics elevates the risk value beyond the model’s average prediction. Specifically, the patient was forecasted to have a fatal outcome due to labureanitrogen, lablactate, and aki_stage, yet mitigating the risk were blue traits like weight and age. Conversely, Figure 2C portrays a lower-risk instance where the patient’s risk profile is diminished by blue attributes such as labsodium, body weight, and output_amount, resulting in a notable risk reduction to *f*(*x*) = −3.60.

The figure illustrates a distinct hierarchical structure regarding the importance of features. The most critical features exert the greatest influence on prediction outcomes. The high-importance features, identified as the primary decisive variables, include arterial partial pressure of oxygen, SAPS II score, and AKI stage. The mean (SHAP) of SAPS II, AKI stage and oxygenation index ranks among the top. These features exhibit significant positive changes in SHAP values, indicating their substantial contribution to risk prediction. Moderately important features, categorized as auxiliary predictive variables, encompass weight, age, red blood cell distribution width, and blood sodium, among others. While these variables contribute moderately to the model and play a role in risk assessment, their impact is less pronounced than that of the core variables associated with disease severity and renal function. The low-importance characteristics include lactic acid, urine volume, and blood urea nitrogen. The SHAP analysis results reveal that oxygenation status, disease score, and renal function status are the core variables predicted by the model, contributing most significantly to the prediction of adverse outcomes. Although the remaining variables possess some clinical reference value, their predictive effects within this model are relatively limited.

### External verification

We validated an external cohort recruited from the General Hospital of Ningxia Medical University (Ningxia, China) between 2023 and 2025. The patients in the external validation cohort were older than those in the training cohort. Furthermore, the external validation cohort contained fewer patients with AKI stage III compared to the training cohort. Among all models evaluated in the external validation, XGBoost demonstrated the highest performance, with an AUC of 0.8799. Additionally, we compared the DOC curves of the five models in the validation sequence, confirming that XGBoost exhibited the best overall performance (Figure 4).

## Discussion

Sepsis, a leading cause of morbidity and mortality among critically ill patients, presents a significant challenge for the medical community. Within this intricate pathological condition, acute kidney injury (AKI) is one of the most prevalent and severe complications. The complexity and substantial heterogeneity of sepsis impose numerous limitations on current treatment strategies and clinical decision-making processes. These challenges not only impact patient prognosis but also underscore the urgent need for the development of more effective predictive models. Therefore, this study aims to improve the risk stratification of death in patients with S-AKI by applying advanced machine learning (ML) techniques, rather than the prediction of AKI onset. This approach aims to facilitate timely and effective interventions, ultimately improving clinical outcomes for patients.

Our research findings indicate that Machine Learning (ML) models, particularly the XGBoost model, can effectively predict the risk of Acute Kidney Injury (AKI) based on various clinical and laboratory parameters. Moreover, by incorporating the clinical phenotypic characteristics of sepsis, the ML model further underscores its potential to influence clinical decision-making. Specifically, the Area Under the Curve (AUC) of the model was 0.8799, demonstrating its robust predictive performance in distinguishing between patients at risk for AKI and those not at risk. This study not only highlights the significance of early identification and intervention but also illustrates the essential role of advanced computational methods in enhancing patient outcomes in intensive care settings.

This study underscores the critical role of multiple biomarkers in predicting Acute Kidney Injury (AKI) in patients with sepsis, emphasizing the significance of the Simplified Acute Physiology Score II (SAPS II). The analysis examines the influence of SAPS II, AKI stage, and serum sodium levels on predictive outcomes. SAPS II evaluates disease severity based on various physiological parameters and has been validated as a predictor of mortality in severely ill patients. Consequently, it holds substantial value for risk stratification in sepsis-related AKI (21). The findings indicate that a higher SAPS II score correlates with an elevated mortality rate, suggesting that this score is instrumental in identifying high-risk patients with poor prognoses in clinical settings, We propose risk strata based on clinically meaningful probability thresholds to support intensified monitoring and timely renal-support consideration.

Additionally, studies have shown that the severity of AKI plays a critical role. Patients at advanced AKI stages exhibit a worse prognosis, with higher mortality rates and an increased need for renal replacement therapy (22). Furthermore, serum sodium levels serve as crucial prognostic indicators. Deviations in sodium levels are closely linked to elevated mortality rates and AKI progression. This aligns with existing literature suggesting that electrolyte imbalances, particularly sodium imbalances, can exacerbate renal damage and complicate the clinical management of sepsis patients (23). Incorporating these biomarkers into clinical settings is anticipated to improve the early identification of high-risk AKI patients, facilitate prompt interventions, and ultimately enhance outcomes. The study underscores the importance of these genetic and biochemical markers in elucidating the pathogenesis of sepsis-induced AKI, thereby aiding in refining patient management protocols in intensive care environments.

The research findings indicate that various pathways related to oxygenation status, disease severity scores, and renal function are crucial in the onset and progression of acute kidney injury (AKI) in patients with sepsis. Specifically, the analysis reveals that patients exhibiting higher Sequential Organ Failure Assessment (SOFA) scores and Acute Physiology and Chronic Health Evaluation II (APACHE II) scores experience significantly poorer renal outcomes. This underscores the importance of these two scoring systems in predicting the severity of AKI. Additionally, the research demonstrates that phenomena associated with oxygenation impairment, such as reduced partial pressure of oxygen in the renal medulla, are significantly correlated with the decline of renal function in patients with sepsis.

This discovery aligns with prior research, underscoring the pivotal role of renal hypoxia in the development of sepsis-induced AKI, exacerbating renal cell injury and inflammatory reactions, thus driving disease progression (24). The delineation of these pathways suggests that interventions aimed at enhancing renal oxygenation levels and optimizing fluid balance may yield therapeutic benefits. These approaches can aid in restoring renal function in sepsis patients, consequently reducing mortality rates. Moreover, the research underscores the importance of ongoing monitoring of renal function in critically ill individuals, as prompt identification of AKI enables timely interventions and enhances clinical outcomes (25). In summary, the integration of these findings into clinical settings is anticipated to mitigate the elevated incidence and fatality rates associated with sepsis-induced AKI in this demographic, thereby fostering more efficient treatment protocols.

Analysis of immune-related indicators in sepsis patients indicated notable abnormalities in white blood cell count and lactate levels, suggesting a significantly activated inflammatory response closely linked to acute kidney injury (AKI). A study involving 16,800 patients demonstrated that deceased patients had higher white blood cell counts and lactate levels compared to survivors, highlighting the pivotal role of the inflammatory response in sepsis-related AKI pathogenesis. Excessive inflammation may lead to renal dysfunction and subsequent multiple organ failure. Elevated lactate levels are commonly indicative of tissue hypoperfusion and metabolic acidosis, reflecting the severity of the patient’s inflammatory response and tissue perfusion impairment. Previous studies have indicated that hematological characteristics, including platelets and red cell distribution width (RDW), may offer valuable prognostic information for ICU-acquired acute kidney injury (ICU-AKI) (26). In this study, indicators such as RDW contributed significantly to SHAP and feature selection, suggesting their potential utility in risk stratification. These findings underscore the importance of early identification and management of inflammatory responses in sepsis to mitigate AKI risk and enhance patient outcomes. A comprehensive comprehension of the interplay between immune dysregulation and renal injury is anticipated to establish a theoretical foundation for therapeutic approaches targeting inflammatory responses, ultimately improving the prognosis of critically ill patients (27–33).

This study has several limitations that must be acknowledged. Firstly, the research data is derived solely from a single database, potentially introducing selection bias and constraining the generalizability of the findings. The absence of multi-center validation somewhat diminishes the model’s utility across various patient cohorts and diverse clinical environments. Secondly, this study mainly evaluated the discrimination based on AUC and did not systematically report calibration indicators (such as calibration curves and Brier scores), which limited the direct interpretation of probability prediction in clinical practice. Due to the limitations of the external cohort probability output and resampling process, the calibration curve and Brier score were not reported in this study. They will be supplemented in the future in the multi-center extended validation. Furthermore, these clinical and biochemical markers may help characterize disease severity and inform risk stratification in S-AKI. Subsequent studies should prioritize supplementing calibration evaluation and recalibration analysis in multi-center external validation to assess its clinical application value.

## Conclusion

In conclusion, this study has identified several key predictors of acute kidney injury (AKI) in a sizable cohort of sepsis patients. Notably, the XGBoost model demonstrates superior performance in terms of accuracy, sensitivity, and other metrics. Furthermore, the findings underscore the crucial role of significant clinical parameters like the SAPS II score and AKI stage in risk assessment. The integration of these machine learning models into clinical practice holds promise for enhancing early and precise risk detection, facilitating prompt interventions, and ultimately enhancing patient outcomes. Ongoing exploration and validation of these models across diverse regions and clinical contexts are essential to ensure their reliability and widespread adoption in routine clinical decision-making.

## Data Availability

The original contributions presented in the study are included in the article/supplementary material, further inquiries can be directed to the corresponding author.
